# Influence of Stress on Electronic and Optical Properties of Rocksalt and Wurtzite MgO–ZnO Nanocomposites with Varying Concentrations of Magnesium and Zinc

**DOI:** 10.3390/nano12193408

**Published:** 2022-09-28

**Authors:** Yin-Pai Lin, Sergei Piskunov, Laima Trinkler, Mitch Ming-Chi Chou, Liuwen Chang

**Affiliations:** 1 Institute of Solid State Physics, University of Latvia, 8 Kengaraga Str., LV-1063 Riga, Latvia; 2 Center of Crystal Research, Department of Materials and Optoelectronic Science, National Sun Yat-Sen University, 70 Lienhai Rd., Kaohsiung 80424, Taiwan

**Keywords:** rocksalt Mg_1−x_Zn_x_O, wurtzite Zn_1−x_Mg_x_O, optical properties, pressure, MgO–ZnO alloys

## Abstract

The structural, electronic and optical properties of stressed MgO–ZnO nanocomposite alloys with concentrations of Zn and Mg varying from 0.125 to 0.875 were studied using ab initio simulations. Two crystal structures are considered for the initial MgO–ZnO alloys: the rocksalt Mg${}_{1}{}_{-}{}_{x}$Zn${}_{x}$O and wurtzite Zn${}_{1}{}_{-}{}_{x}$Mg${}_{x}$O phases. For rocksalt Mg${}_{1}{}_{-}{}_{x}$Zn${}_{x}$O, the optimized structures are stable at pressures below 10 GPa. The larger the Mg concentration and pressure, the wider the $E_{g}$ of the rocksalt phase. In contrast, the optimal geometries of wurtzite Zn${}_{1}{}_{-}{}_{x}$Mg${}_{x}$O reveal a diversity of possibilities, including rocksalt, wurtzite and mixed phases. These effects lead to the fact that the optical properties of wurtzite Zn${}_{1}{}_{-}{}_{x}$Mg${}_{x}$O not only demonstrate the properties of the wurtzite phase but also indicate the optical features of the rocksalt phase. In addition, mixed phases of Zn${}_{1}{}_{-}{}_{x}$Mg${}_{x}$O simultaneously provide the characteristics of both wurtzite and rocksalt phases with the same structures in different dielectric matrices.

## 1. Introduction

Zinc oxide (ZnO) is one of the most promising metal oxide materials in the industry and other engineering fields for optical utilization based on its sensing [1], antibacterial [2] and water-splitting [3] properties. The synthesis of ZnO structures by a variety of chemical and physical processes has been studied [4]. From the viewpoint of the optical spectrum, the band gap ($E_{g}$) of ZnO is approximately from 3.3 to 3.4 eV, which allows for optical applications in the ultraviolet range [5]. Magnesium oxide (MgO) is known as a typical wide band gap material with strong ionic bonding [6]. As for its bulk structure, MgO has a cubic form with an $E_{g}$ of up to 7.7 eV [7]. Both the wide band gap material and the nonlinear susceptibility of MgO have numerous applications in many fields, including photosensors [8], photocatalysts [9] and nonlinear optical devices [6,10]. Both MgO and ZnO often contain point defects, which determine and significantly affect their functional properties [11,12,13,14].

The nanocomposites or alloys of MgO and ZnO (ZMO) facilitate $E_{g}$ engineering, including photosensors, photocatalysts, photodetectors and solar cell devices [15,16,17,18], to achieve the properties of a wide optical response range from 3.3 to 7.8 eV [4,19]. Under ambient conditions, MgO has rocksalt (RS) crystal structures, and ZnO has those of wurzite (WZ). With the development of synthesis, RS Mg${}_{1}{}_{-}{}_{x}$Zn${}_{x}$O and WZ Zn${}_{1}{}_{-}{}_{x}$Mg${}_{x}$O have been produced in recent years [4,19,20,21,22,23,24]. In addition, both controlling the concentration of ZMO and applying external pressure have led to $E_{g}$ engineering. Indeed, the factor of pressure may induce variations in the phase transitions and lattice constants. MgO-ZnO alloys have been reported in ordered ground-state structures at pressures above approximately 6.5 GPa, and the structures are dependent on high pressures, including *Pm-3m, I4/mmm, P4/mmm, I41/amd, C2/m, Cmcm, I4/m* and *R-3* [25], yet few studies of ZMO models that simultaneously consider the parameters of concentration for magnesium and zinc and the pressure exist.

From the experimental measurements, detecting the $E_{g}$ of ZMO has commonly relied on the methods of spectroscopic ellipsometry, optical absorption and photoluminescence, which may also be theoretically predicted by the frequency-dependent dielectric function via the density functional theory within the linear response theory and the Bethe–Salpeter equation for excitonic effects [20,26,27,28,29,30]. However, the optical properties of the ZMO models involving concentration and pressure remain unclear as the electronic structures inclusive of the density of states and frequency-dependent dielectric function have not been thoroughly investigated. The combination of ZnO and MgO under pressure may trigger the distinction of lattice constants or even change the original crystallographic, which will reorganize the locations of the valence and conduction bands. The new formations of the density of states absolutely change the behavior of the interband transition and may result in a new optical spectrum. The RS and WZ phases of the ZMO models with identical conditions for the concentration under pressure have not been studied simultaneously.

Specifically, the TEM images show the apparent interface of ZnO/MgO, grain boundaries and defects as well as indicate that the interface also has a tremendous influence on the electronic and optical properties [31,32]. From theoretical predictions of the optical properties of a supercell [20,30], the optical spectrum reflects some characteristic peaks in comparison with the experimental absorbance. In addition, the strict computational cost requirements of constructing interface systems for the excitonic effect cannot be avoided, which must be taken into account to estimate the exact absorption onset for both ZnO and MgO [33]. Hence, in terms of experiments, the interface systems are closer to the real atomic structures [31,32], but an investigation of this relationship and structural factor are beyond the current purposes of controlling the concentration and external pressure in this study.

In this paper, the structural, electronic and optical properties have been calculated with respect to the RS and WZ ZMO models to investigate the absorption spectrum under stress. The computational methods, as well as the RS and WZ ZMO models, are described in Section 2. Section 3 presents the effects of the varying concentrations under external pressure on the frequency-dependent absorption coefficient of the RS and WZ ZMO models. Finally, we conclude with the achieved results in Section 4.

## 2. Computational Methods and Models

### 2.1. Methodology

The density functional theory (DFT) code of GPAW [34,35] was utilized to calculate the electronic structures and optical properties, in which the electronic wave functions are based on the projector-augmented wave method and the atomic simulation environment (ASE) [36,37] for geometric optimizations. The electronic configurations of magnesium, oxygen and zinc were 2s${}^{2}$2p${}^{6}$3s${}^{2}$, 2s${}^{2}$2p${}^{4}$ and 3d${}^{10}$4s${}^{2}$ for the valence electrons, respectively. By using the Perdew–Burke–Ernzerhof (PBE) [38] functionals, the geometric parameters of the ZMO models were optimized at pressures from 0 GPa to 10 GPa in 2 GPa increments with the Broyden–Fletcher–Goldfarb–Shanno (BFGS) algorithm until the maximum force of the atoms was below 0.05 eV/Å. The plane-wave cutoff energy was set to 500 eV, and the convergence criteria were considered to be the default values. The Monkhorst–Pack scheme with a k-point of 8 × 8 × 8 was used to sample the Brillouin zone during the relaxations and self-consistent field (SCF) iterations. The DFT calculations were operated using the PBE and the Gritsenko–van Leeuwen–van Lenthe–Baerends functional with the solid-state modification (GLLBSC) [39] functionals. To overcome the underestimated band gap ($E_{g}$), the localized quantities of electrons were interpreted via the Hubbard DFT+U term [40], of which the corrections of the on-site Coulomb U${}_{p,Mg}$ and U${}_{d,Zn}$ were 0.7 [41] and 10 [5] eV, respectively.

To calculate the optical response, the linear response theory (LR) and Bethe–Salpeter equation (BSE) were applied to predict the frequency-dependent dielectric function ϵ(ω) with and without considering the excitonic effects, respectively. For the LR ϵ(ω), a random phase approximation (RPA) was utilized, and the plane-wave energy cutoff for evaluation of the dielectric matrix was determined to be 100 eV. For the BSE ϵ(ω), the convergence of the optical spectrum [30] was calculated by five valence and five conduction bands. Moreover, the plane-wave energy cutoff of the BSE ϵ(ω) was 50 eV for the dielectric matrix. To gain more insight into the optical absorption, the complex dielectric function (ϵ = $\epsilon_{Re}$ + i$\epsilon_{Im}$) was further expressed by the absorption coefficient ($\alpha_{abs}$) using the relation from [42], which is as follows:

$$\alpha_{abs}=\sqrt{2}\omega {\left(\sqrt{\epsilon_{Re}^{2}\left(\omega \right)+\epsilon_{Im}^{2}\left(\omega \right)}-\epsilon_{Re}\left(\omega \right)\right)}^{1/2}.$$

Other software and packages used in this study are as follows: VESTA software [43], NumPy [44] and Matplotlib [45] were used to illustrate the atomic environments, analyze data and generate the figures, respectively.

### 2.2. ZMO Models

**Figure 1:**
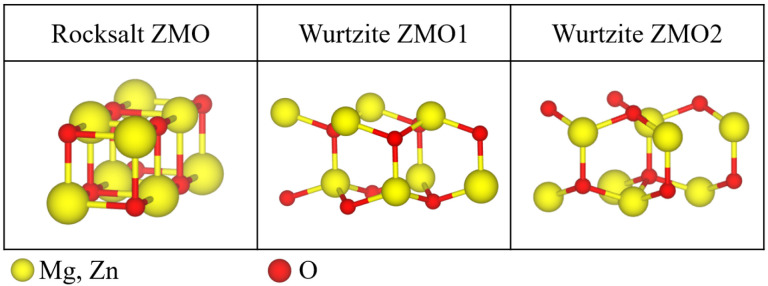
Supercells of 2 × 1 × 1 rocksalt (RS) Mg${}_{1}{}_{-}{}_{x}$Zn${}_{x}$O and 2 × 2 × 1 wurtzite (WZ) Zn${}_{1}{}_{-}{}_{x}$Mg${}_{x}$O for ZMO models. The first column is the atomic structure of RS Mg${}_{1}{}_{-}{}_{x}$Zn${}_{x}$O. The second and third columns are the geometries of WZ Zn${}_{1}{}_{-}{}_{x}$Mg${}_{x}$O for two different unit cells. The yellow spheres are either magnesium (Mg) or zinc (Zn); the red spheres represent oxygen.

Two crystallographic sets were built for the ZMO models. One was the RS phase based on pure MgO crystal of a cubic structure; the other was the WZ phase based on pure ZnO crystal of a hexagonal structure. To compare the different phases with the same concentration, eight oxygen atoms and eight summations of atoms including magnesium and zinc were used to construct the ZMO models, which were the supercell (SC) 2 × 1 × 1 for RS Mg${}_{1}{}_{-}{}_{x}$Zn${}_{x}$O and the SC 2 × 2 × 1 for WZ Zn${}_{1}{}_{-}{}_{x}$Mg${}_{x}$O, respectively. Figure 1 shows the schematic structures for the RS and WZ phases. When the ZMO models were obtained from the DFT calculations with or without the pressure factors, one of the challenges was that the optimization process for the WZ phases can trigger phase transitions during the relaxations. Due to the sensitivity of lattice constants under pressure, there was no constraint during the optimizations. Indeed, the optimized WZ ZMO models do tend to the RS phase and mixed phases during the relaxations. Therefore, two different unit cells of WZ phases were considered for the ZMO models in this work. As the positions of the configurations for the substituted atoms with the same concentration as in the ZMO models would not cause a big discrepancy in the electronic and optical properties, one special configuration was considered for a varied concentration with the substituted atoms distributed homogeneously in the ZMO models [20].

## 3. Results and Discussion

### 3.1. Structural Properties and $E_{g}$ for RS Mg${}_{1}{}_{-}{}_{x}$Zn${}_{x}$O

**Figure 2:**
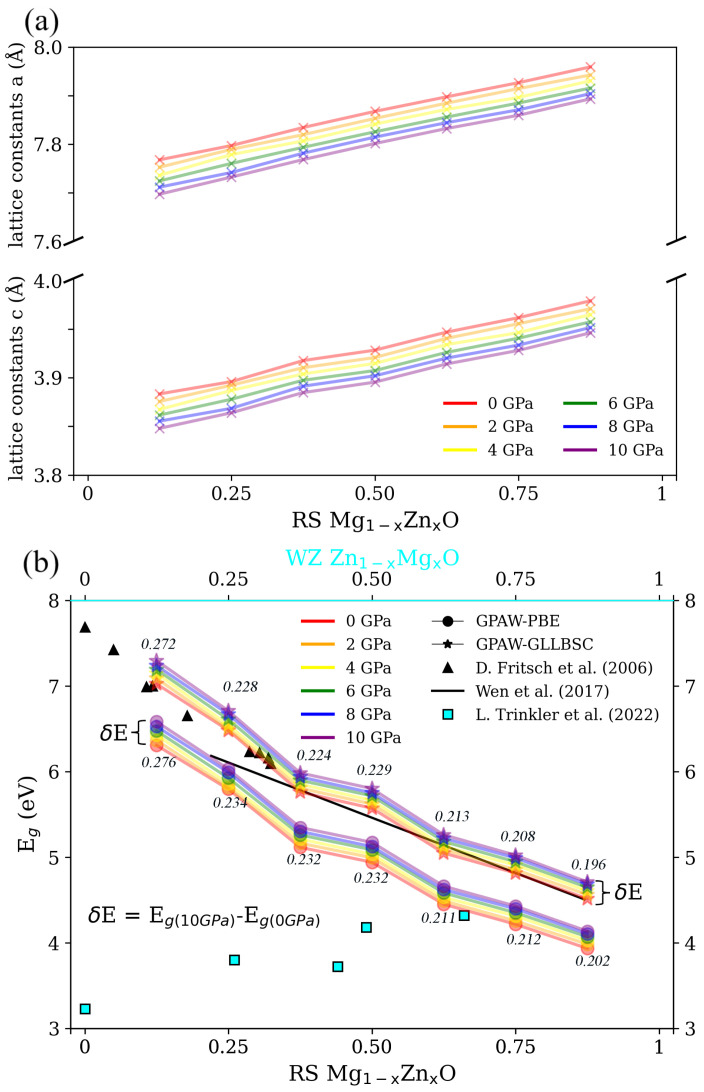
(**a**) The lattice constants and (**b**) band gap ($E_{g}$) of rocksalt (RS) Mg${}_{1}{}_{-}{}_{x}$Zn${}_{x}$O for x = 0.125, 0.25, 0.375, 0.5, 0.625, 0.75 and 0.875 as well as the pressures ranging from 0 to 10 GPa. Subfigure (**a**) presents the lattice constants of the *a* (upper) and *c* (bottom) axes. In Subfigure (**b**), the black triangles and cyan squares are the experimental $E_{g}$ of the RS ZMO alloys [48] and WZ ZMO epilayers [4], respectively. The black line is the fitted result of $E_{g}$ ranging from x = 0.22 to 0.87 according to RS Mg${}_{1}{}_{-}{}_{x}$Zn${}_{x}$O = 4.17 + 2.58(1 − x) eV [19]. The circle and star symbols are the theoretical $E_{g}$ of the PBE and GLLBSC functionals, respectively. The colors red, orange, yellow, green, blue and purple correspond to 0, 2, 4, 6, 8 and 10 GPa. The difference in the theoretical $E_{g}$ (δE) between 10 and 0 GPa is marked. The top label of cyan color corresponds to the expression of wurtzite (WZ) Zn${}_{1}{}_{-}{}_{x}$Mg${}_{x}$ for the experimental concentration in Subfigure (**b**). The detailed $E_{g}$ in Appendix A of Table A1 corresponds to the values in Subfigure (**b**).

The lattice constants and $E_{g}$ calculated for the different concentrations and pressures of RS Mg${}_{1}{}_{-}{}_{x}$Zn${}_{x}$O are shown in Figure 2. Initially, the crystallographic properties of the RS ZMO models are well sustained during the optimization. With the increments of the Zn concentration and pressure, there is no phase transition in the RS ZMO models. Although the angle of the RS models (α, β, γ) slightly decreases after relaxations, the differences are smaller than 0.001 of a degree. Notably, the experimental lattice constants of the pure RS MgO bulk are around 4.2 Å [46]. However, the optimal lattice constants of the RS ZMO models obviously decrease, as shown in Figure 2a. Nevertheless, the Hubbard U parameter is helpful for improving the underestimated $E_{g}$. However, the on-site Coulomb interactions of the *p* and *d* localized electrons also induce the decreasing lattice constants of the RS and WZ phases as compared to the experiments due to the Hubbard corrections of magnesium and zinc [47]. The denser the concentration of zinc, the larger the values of the lattice constants. Meanwhile, the high external pressure shrinks the lattice constants for both the *a* and *c* axes.

Subsequently, the experimental $E_{g}$ is utilized to test the precision of the RS ZMO models in Figure 2b. For the RS ZMO, Fritsch et al. reported an $E_{g}$ with a low concentration of zinc [48], and Wen et al. also fitted the data between the concentration and $E_{g}$ based on the experiments in [19] with a wider ratio of the concentration. In addition, Trinkler et al. measured the WZ ZMO epilayers with different concentrations [4]. In brief, the experiments demonstrate that the $E_{g}$ of the RS ZMO can be individually adjusted in between the ranges from 4.5 to 7.8 eV and from 3.2 to 4.4 eV. On the basis of the experimental $E_{g}$, the RS ZMO models with an increasing zinc concentration and pressure are in agreement with the RS phases, especially the results obtained by means of the GLLBSC functional. Meanwhile, the discrepancy between the higher and lower pressure is the variation in $E_{g}$. In brief, a decreasing concentration of magnesium and a higher external pressure bring about a reduction in the lattice constants, which also triggers a wider $E_{g}$. At the same time, a denser concentration of zinc reduces the difference in the $E_{g}$ between the higher and lower pressure.

### 3.2. Structural Properties and $E_{g}$ for WZ Zn${}_{1}{}_{-}{}_{x}$Mg${}_{x}$O

**Figure 3:**
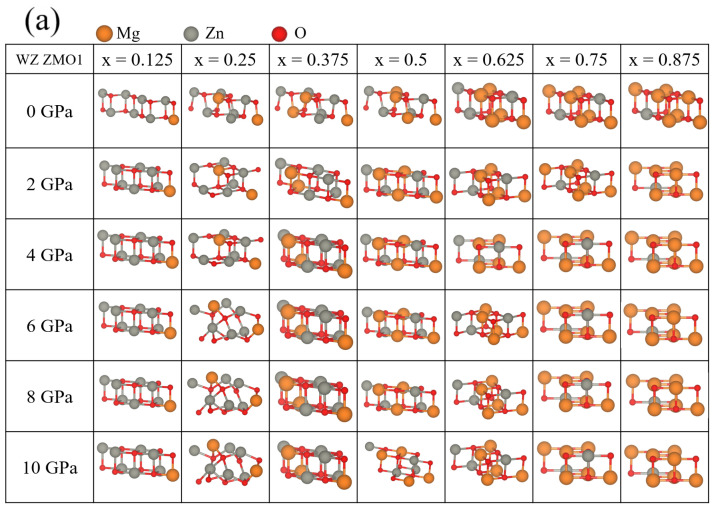

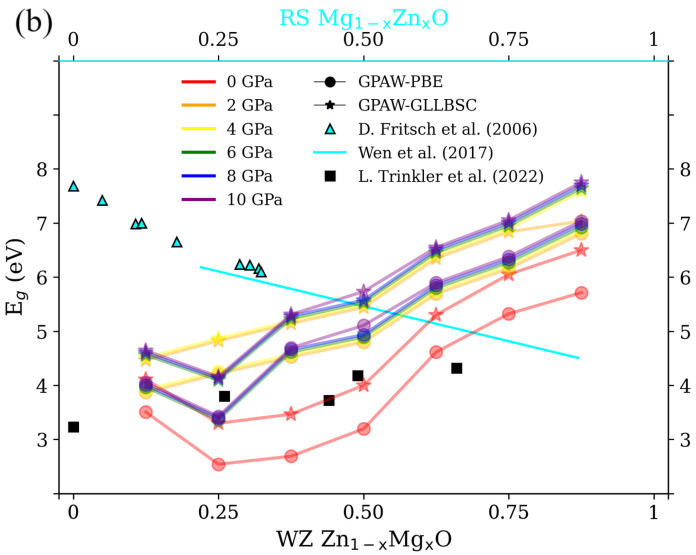
(**a**) Optimized structures and band gap ($E_{g}$) of wurtzite (WZ) Zn${}_{1}{}_{-}{}_{x}$Mg${}_{x}$ for the WZ ZMO1 model in Figure 1 with x = 0.125, 0.25, 0.375, 0.5, 0.625, 0.75 and 0.875 as well as the pressures from 0 GPa to 10 GPa in 2 GPa increments. In Subfigure (**a**), the colors orange, grey and red correspond to magnesium, zinc and oxygen, respectively. The experimental data of the line and triangles for rocksalt (RS) Mg${}_{1}{}_{-}{}_{x}$Zn${}_{x}$O are colored cyan; the black squares are the experimental results of WZ Zn${}_{1}{}_{-}{}_{x}$Mg${}_{x}$O. The other symbols, colors and legends of Subfigure (**b**) are identical to the descriptions in Figure 2b. The top label of cyan color corresponds to the expression of rocksalt (RS) Mg${}_{1}{}_{-}{}_{x}$Zn${}_{x}$O for experimental concentration. The $E_{g}$ detailed in Appendix A of Table A2 corresponds to the values in Subfigure (**b**) [4,19,48].

In contrast, the relaxed structures and $E_{g}$ for the WZ ZMO models reveal more complicated results than the RS ZMO models. In the optimized structures of both WZ ZMO models, mixed phases can be found, which simultaneously have the crystallographic properties of the RS and WZ phases. For the WZ ZMO1 models, the optimized structures with an increasing concentration of magnesium and pressure are mostly inclined to the RS phase in Figure 3a. Only seven optimal geometries indicate that the included angles of the structures are not obviously orthogonal in the concentrations of x = 0.25 with 0, 6, 8 and 10 GPa as well as x = 0.125, 0.375 and 0.5 with 0 GPa. According to the experimental, most $E_{g}$ of the WZ ZMO1 models meet the distributions from 4.5 to 7.8 eV, except those seven ZMO1 cases. At first glance, the situations of x = 0.125, 0.375 and 0.5 with 0 GPa tend to the RS phases. However, the $E_{g}$ values (red stars and circle at x = 0.125, 0.25, 0.375 and 0.5) are apparently smaller than those at the same concentration with different pressure. The tiny discrepancy in the included angle presents the characteristic $E_{g}$ of the WZ ZMO even if the appearance of the lattice tends to the composition of the RS phase. In addition, the external pressure is significantly positive for the phase transition from the WZ to RS phase. In the ZMO1 models, most of the cases with external pressure approach the RS phases. Although the $E_{g}$ of the concentration x = 0.25 with the increments of pressure reveals the phase transitions WZ–RS–WZ, this phenomenon may be triggered by the asymmetrical structures of the doped atoms.

**Figure 4:**
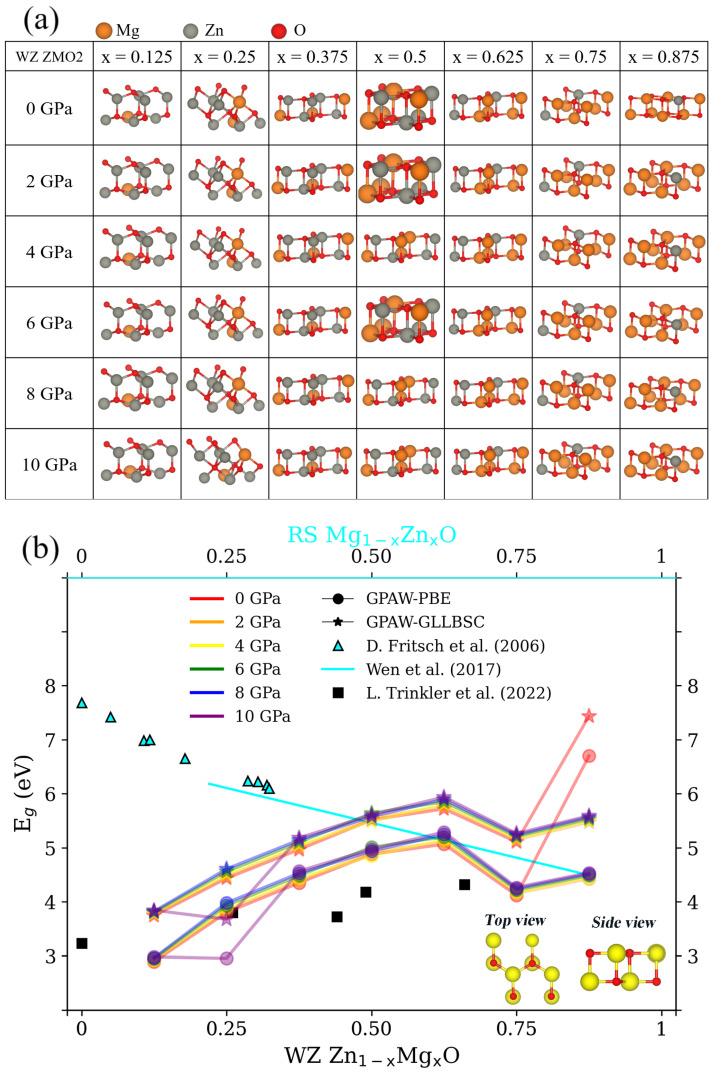
(**a**) Optimized structures and band gap ($E_{g}$) of wurtzite (WZ) Zn${}_{1}{}_{-}{}_{x}$Mg${}_{x}$ for the WZ ZMO2 model in Figure 1 with x = 0.125, 0.25, 0.375, 0.5, 0.625, 0.75 and 0.875, as well as the pressures from 0 GPa to 10 GPa in 2 GPa increments. The symbols, colors and legends of Subfigure (**a**,**b**) are identical to the descriptions in Figure 3a,b, respectively. The insets of Subfigure (**b**) are the top and side views of the structures of x = 0.75 and 0.875 in Subfigure (**a**), except for the structure of x = 0.875 within 0 GPa. The yellow spheres are either magnesium (Mg) or zinc (Zn); the red spheres represent oxygen. The $E_{g}$ detailed in Appendix A of Table A3 corresponds to the values in Subfigure (**b**) [4,19,48].

Considering the other WZ ZMO model, the ZMO2 structures reveal more varied consequences than the ZMO1 models and are shown in Figure 4a. First, the concentration of x = 0.125 sustains the features of the hexagonal structure for the WZ phase within the increasing pressure. Meanwhile, the $E_{g}$ of x = 0.125 is in agreement with the experiments of the WZ phase in Figure 4b. Second, the structures tend to the RS phases when the concentrations were 0.375, 0.5 and 0.625 within different pressures. Consequently, the $E_{g}$ of x = 0.375, 0.5 and 0.625 with and without pressure fulfill the distribution of the experimental RS $E_{g}$. Namely, the WZ Zn${}_{1}{}_{-}{}_{x}$Mg${}_{x}$ for x = 0.375, 0.5 and 0.625 correspond to the RS MgO for Mg${}_{1}{}_{-}{}_{x}$Zn${}_{x}$O for x = 0.625, 0.5 and 0.375, respectively. The relationship between the theoretical results and experimental reference is a mirror-image relationship for the reference point of x = 0.5. Third, the insets of Figure 4b illustrate the top view of the hexagon and the side view of the cube for the WZ ZMO2 at x = 0.75 and 0.875, except for the case of x = 0.875 within 0 GPa, which still shows the feature of a cubic rocksalt appearance. Due to the hexagonal distributions, the $E_{g}$ of the WZ ZMO2 at x = 0.75 and 0.875 shows an obvious reduction in comparison to the same concentration in Figure 3b. As for the concentration of x = 0.25, the phase transition was induced at a pressure of 10 GPa based on the decreasing $E_{g}$ in Figure 4b. In summary, the RS ZMO models are in agreement with the experimental $E_{g}$. A denser concentration of magnesium and higher pressure cause a wider $E_{g}$ due to the reduction in lattice constants. In contrast, for the WZ ZMO models, it is difficult to maintain the characteristics of the WZ phase within the different concentrations and various external pressures.

### 3.3. Density of States

**Figure 5:**
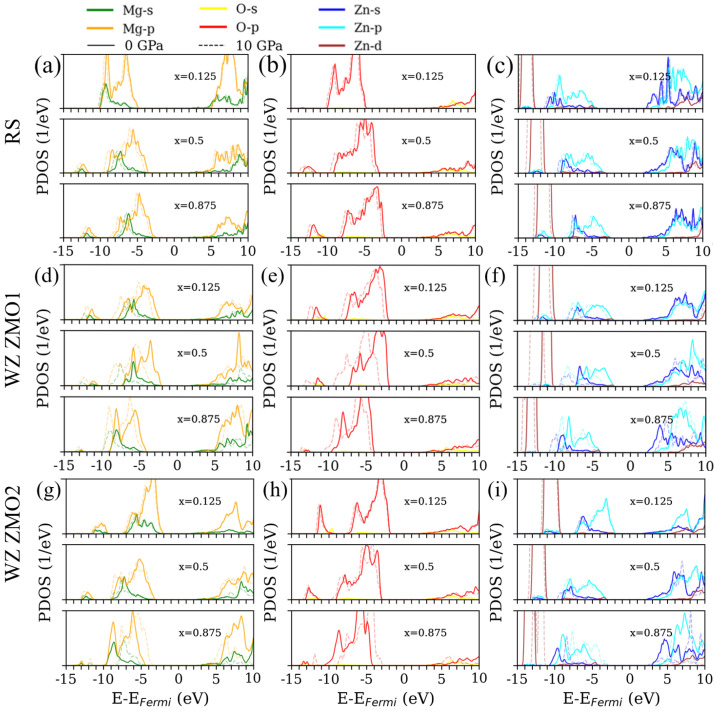
Partial densities of states (PDOS) of rocksalt (RS) Mg${}_{1}{}_{-}{}_{x}$Zn${}_{x}$O and wurtzite (WZ) Zn${}_{1}{}_{-}{}_{x}$Mg${}_{x}$O at different concentrations (x = 0.125, 0.5 and 0.875). The RS ZMO (**a**–**c**), WZ ZMO1 (**d**–**f**) and WZ ZMO2 (**g**–**i**) are shown in Figure 1. The first, second and third columns correspond to magnesium, oxygen and zinc, respectively. The upper, medium and bottom panels are related to the concentrations of x = 0.125, 0.5 and 0.875 in each sub-figure. The colors green, orange, yellow, red, blue, cyan and brown correspond to the Mg–*s*, Mg–*p*, O–*s*, O–*p*, Zn–*s*, Zn–*p* and Zn–*d* states, respectively. The solid and dashed lines individually present the 0 and 10 GPa. The Fermi level is set to zero. The detailed PDOS of the ZMO models for x = 0.125, 0.5 and 0.875 with the pressure from 0 GPa to 10 GPa in 2 GPa increments can be found in Appendix B, which features Figure A1 for the RS models, Figure A2 for the WZ ZMO1 models and Figure A3 for the WZ ZMO2.

The calculated partial densities of states (PDOS) for the RS Mg${}_{1}{}_{-}{}_{x}$Zn${}_{x}$O are shown in Figure 5a–c. Because of the accurate $E_{g}$, the optical absorption and electronic structures are calculated by the GLLBSC functionals with DFT+U. Thus, only three concentrations (x = 0.125, 0.5 and 0.875) will be discussed in order to simplify the illustration. Initially, the increasing pressure causes the DOS curves to shift to lower energies, and the valence states are more obvious than the states forming the conduction band. Due to the reduction in lattice constants with the higher pressure, a stronger interaction of the localized electrons causes the $E_{g}$ to be slightly wider. Second, the increasing concentration of zinc causes the valence band maximum (VBM) to become closer to the Fermi level. Because the Zn–*s* states decrease around the conduction band minimum (CBM), the influence of the Zn–*p* states is gradually enhanced. In addition, the Mg–*s* and Mg–*p* states occupy the higher conduction bands in comparison to the Zn–states. The interactions between Zn and O grow continuously stronger with the increments of zinc concentration, which is helpful for the formation of bonding and anti-bonding between the Zn–*p* and O–*p* [30,49] and allows the $E_{g}$ to be narrower.

The calculated partial densities of states (PDOS) for the WZ ZMO1 and WZ ZMO2 are shown in Figure 5d–f and Figure 5g–i, respectively. For the optimized cases of the WZ ZMO1, it can be found that the apparent difference in the phase transition arises from pressure. From the viewpoint of the $E_{g}$ in Figure 3b, the WZ ZMO1 of x = 0.5 under 0 GPa is inclined to the WZ phases. With an external pressure of 10 GPa, the WZ ZMO of x = 0.5 becomes the RS phase in which an obvious shift in the PDOS curves can be observed in comparison with the situation under 0 GPa. In fact, the RS and WZ phases exhibit a difference in energies between the Fermi level and VBM, and the VBM of the WZ phases is usually closer to the Fermi energy. It is worth mentioning that the Hubbard U parameter modifies the positions of occupied Zn–*d* states to the lower energies [50] because of the application of the DFT+U theory. Therefore, there are few states of Zn–*d* around the VBM.

In general, the O–*p* and the Mg–*p* states are mainly occupied around the VBM for both the RS and WZ ZMO models. Around the CBM, the states of zinc affect the variation of the $E_{g}$ due to the hybridizations of the Zn and O states. In addition, the external pressure will mainly cause the valence bands to shift to lower energies. For the electronic structures, the controlling concentration is much more important than the external pressure without considering the phase transitions.

### 3.4. Absorption Coefficient

**Figure 6:**
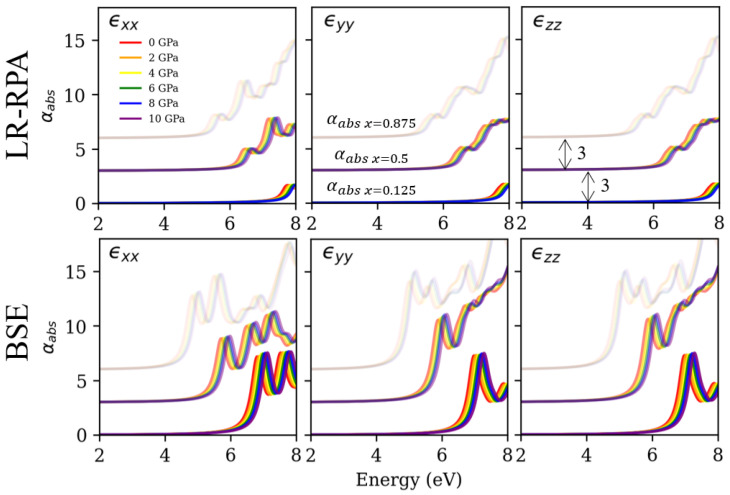
The frequency-dependent absorption coefficient for rocksalt (RS) Mg${}_{1}{}_{-}{}_{x}$Zn${}_{x}$O based on LR ϵ(ω) via the RPA (upper panel) and BSE ϵ(ω) (bottom panel). The first, second and third columns for each sub-figure correspond to the direction of $\epsilon_{xx}$, $\epsilon_{yy}$ and $\epsilon_{zz}$, respectively. The colors red, orange, yellow, green, blue and purple correspond to 0, 2, 4, 6, 8 and 10 GPa. The dark, medium and light gray scaling express the absorption coefficient within the concentrations of 0.125, 0.5 and 0.875, respectively. Moreover, the spectrum with the different concentrations of 0.125, 0.5 and 0.875 is individually adjusted by adding the values of 0, 3, 6 to the y axis.

**Figure A1:**
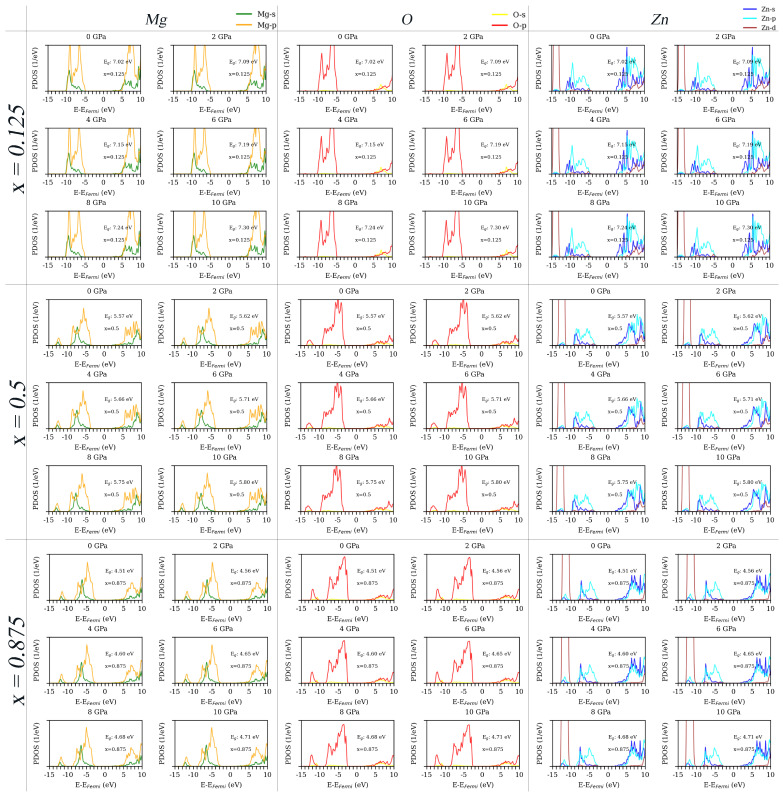
Partial density of states (PDOS) of rocksalt (RS) Mg${}_{1}{}_{-}{}_{x}$Zn${}_{x}$O for RS ZMO models at different concentration (x = 0.125, 0.5 and 0.875) with the pressure from 0 to 10 GPa in 2 GPa increments, which correspond to Figure 5a–c.

In accordance with the electronic structures reported in the previous section, Figure 6 shows the estimated LR $\alpha_{abs}$ via the RPA and BSE $\alpha_{abs}$ for all electronic structures of the RS ZMO models in Figure A1. To start with the excitonic effects, there is a big difference between the LR $\alpha_{abs}$ via the RPA and BSE $\alpha_{abs}$. The interaction between the electrons and holes is utilized to build accurate spectra for RS MgO and WZ ZnO inclusive of the spectral characteristics and absorption onset [33]. These things considered, the LR $\alpha_{abs}$ will be wrongly blue-shifted for the entire spectra, and the first peaks of the absorption onset will be underestimated. Next, the spectra reflect the results of the $E_{g}$ in Figure 2b. An increase in the Zn concentration causes a decrease in the $E_{g}$. Thus, the variation in the absorption onset for the red-shifted spectrum is in agreement with the approximately 3 eV decrement of $E_{g}$ and the increments of the valence bands in Figure 5a–c. Similarly, the external pressure may also affect the spectrum that is blue-shifted around 0.2 eV with an increasing pressure based on the difference in the $E_{g}$ (δE) in Figure 2b. These observations imply that the ratio of Mg and Zn is suitable for realizing major control and that the external pressures are used to modify the minor shift in the $E_{g}$ and spectrum.

**Figure 7:**
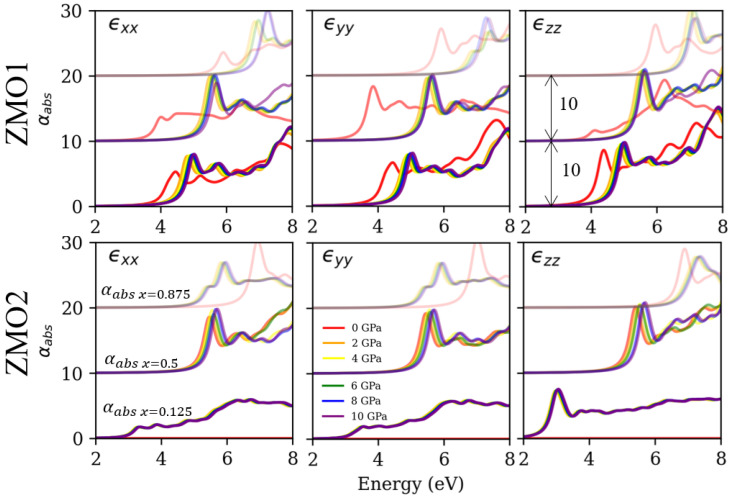
The frequency-dependent absorption coefficient of wurtzite (WZ) Zn${}_{1}{}_{-}{}_{x}$Mg${}_{x}$O based on the BSE ϵ(ω) for the WZ ZMO1 (upper panel) and WZ ZMO2 (bottom panel). The WZ ZMO1 and WZ ZMO2 correspond to the illustrations in Figure 1. The spectrum with different concentrations of 0.125, 0.5 and 0.875 is individually adjusted by adding the values of 0, 10 and 20 to the y axis. The symbols, colors and legends of this figure are identical to the descriptions in Figure 6.

**Figure A2:**
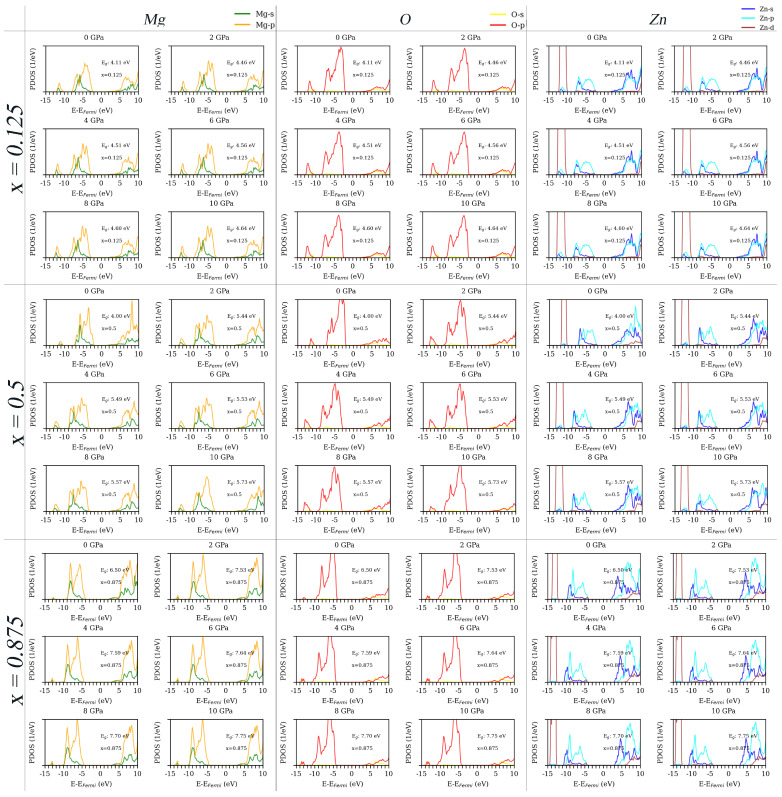
Partial density of states (PDOS) of wurtzite (WZ) Zn${}_{1}{}_{-}{}_{x}$Mg${}_{x}$O for WZ ZMO1 models at different concentration (x = 0.125, 0.5 and 0.875) with the pressure from 0 to 10 GPa in 2 GPa increments, which correspond to Figure 5d–f.

**Figure A3:**
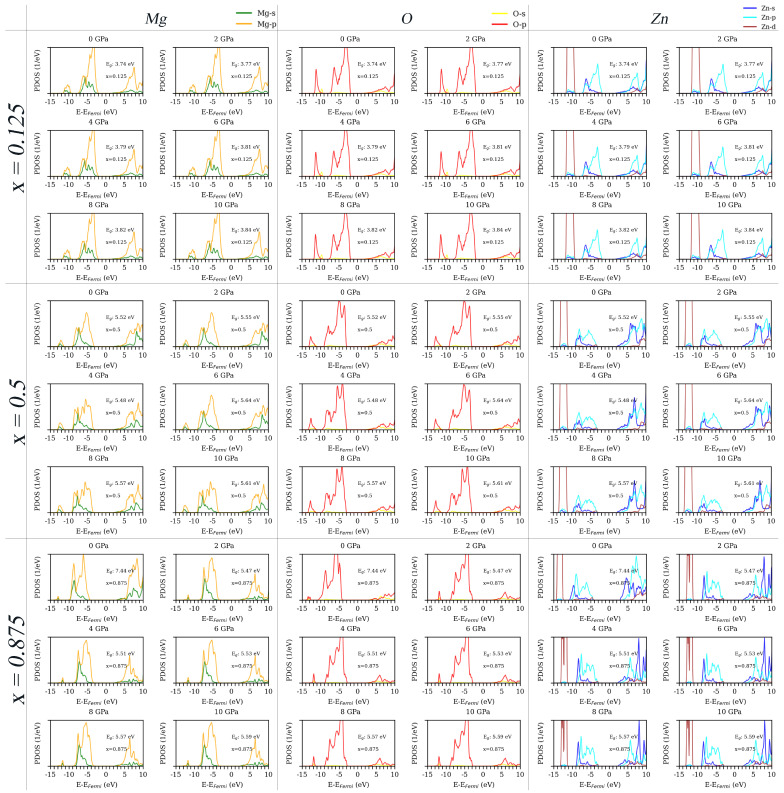
Partial density of states (PDOS) of wurtzite (WZ) Zn${}_{1}{}_{-}{}_{x}$Mg${}_{x}$O for WZ ZMO2 models at different concentration (x = 0.125, 0.5 and 0.875) with the pressure from 0 to 10 GPa in 2 GPa increments, which correspond to Figure 5g–i.

Unlike the spectrum of the RS ZMO models, the phase transitions and mixed phases provide the diverse optical response for the WZ ZMO models in Figure 7, which is based on the electronic structures of the WZ ZMO1 in Figure A2 and WZ ZMO2 models in Figure A3. For the WZ phase, the WZ ZMO2 of x = 0.125 under 0 GPa obviously demonstrates the anisotropic properties in the different directions of the dielectric matrix. Moreover, the absorption onset and first peak are similar to the absorption peak for pure ZnO [5]. As for the pressure that induces the phase transitions, the WZ ZMO1 of x = 0.5 under a pressure from 0 to 10 GPa clearly shows the blue-shifted absorption onset from 4 to 5.5 eV, which reflects the influence on the RS phases of the structural geometries in Figure 3. Among the mixed phases in the same structure, the WZ ZMO2 of x = 0.875 may observe this effect. In Figure 4a, the WZ ZMO2 of x = 0.875 without pressure is the cubic structure, and the $E_{g}$ is up to 7 eV. When the external pressures are considered, the structures of the WZ ZMO2 of x = 0.875 under pressure have hexagonal arrangements in the x and y directions, as well as the cubic style in the z orientation. Therefore, the absorption onset of $\epsilon_{xx}$ and $\epsilon_{yy}$ for the WZ ZMO2 with x = 0.875 under pressure are red-shifted in comparison with the pressure of 0 GPa, while the $\epsilon_{zz}$ are blue-shifted.

Briefly, the optical response of the RS ZMO models indicates uniform results. The absorption onset and first peak locations are above 4 eV, which can be utilized in deep ultraviolet applications. Moreover, the effect of the pressure can perform a minor adjustment to shift the entire spectrum. In contrast, the spectrum of the WZ ZMO models reveals potential for various optical features. For the conventional WZ phases, the absorption spectrum is similar to the WZ ZnO, including the anisotropic absorption and working around the edge of blue light. When the concentration of magnesium and the external pressure are considered, there are two types of phase transitions. One is the RS phase; the other is the mixed phase. For the RS phases, the optical properties are similar to the original RS ZMO models. However, the included angle of the structures is not slightly orthogonal, which causes the $E_{g}$ to be smaller than that of the identical concentration of the RS ZMO models. For the mixed phases of anisotropic absorption, this type of WZ ZMO model not only inherits the properties of the WZ phases but also embodies the large $E_{g}$ of the RS phases. Due to this feature, the mixed phases of the WZ ZMO models may reveal the optical characteristic of both the RS and WZ phases in different directions.

## 4. Conclusions

To sum up, the geometry, electronic structures and optical properties for the RS and WZ phases of the ZMO models under pressure have been investigated with control of the concentrations of Mg and Zn, taking into consideration the effect of pressure. Overall, the varied ratios of Mg and O may achieve a wider adjustment of the $E_{g}$, and the external pressure further tunes the minor control of the $E_{g}$. A denser concentration of zinc reduces the band gap energy and red-shifts the entire absorption spectrum. A higher pressure enlarges the $E_{g}$ but may cause phase transitions and mixed phases. Indeed, the RS ZMO models are more stable than the WZ phases according to our DFT modeling. However, the optimized WZ ZMO models reveal mixed phases, which can include the optical features of RS and WZ with the same structures in different directions. Meanwhile, the positions of the doped atoms induce the anisotropic effect of the dielectric tensor, which is much easier to observe in the WZ ZMO models. This phenomenon provides a diversity of possibilities for designing the optical spectrum. Our theoretical models may provide meaningful references for the development and design of optoelectronic devices from the edge of blue light to deep ultraviolet.

## Data Availability

The raw/processed data required to reproduce these findings cannot be shared at this time as the data also form a part of an ongoing study.

## Appendix A. Band Gap Energy (*Eg*) for Rocksalt Mg_1−x_Zn_x_O and Wurtzite Zn_1−x_Mg_x_O

**Table A1:** Detailed band gap energy ($E_{g}$) of 2 × 1 × 1 rocksalt Mg${}_{1}{}_{-}{}_{x}$Zn${}_{x}$O for varied concentration with external pressure from 0 to 10 GPa in 2 GPa increments corresponding to Figure 2b.

x	0 GPa	2 GPa	4 GPa	6 GPa	8 GPa	10 GPa
	PBE, GLLBSC (eV)					
0.125	6.31, 7.02	6.36, 7.09	6.43, 7.15	6.47, 7.19	6.53, 7.24	6.58, 7.30
0.25	5.79, 6.48	5.82, 6.50	5.86, 6.54	5.92, 6.61	5.99, 6.67	6.03, 6.71
0.375	5.11, 5.76	5.16, 5.81	5.21, 5.85	5.26, 5.90	5.30, 5.94	5.35, 5.98
0.5	4.94, 5.57	4.99, 5.62	5.03, 5.66	5.09, 5.71	5.12, 5.75	5.17, 5.80
0.625	4.45, 5.05	4.49, 5.09	4.53, 5.13	4.58, 5.18	4.62, 5.22	4.66, 5.26
0.75	4.22, 4.81	4.25, 4.85	4.31, 4.91	4.35, 4.95	4.40, 4.99	4.43, 5.02
0.875	3.93, 4.51	3.98, 4.56	4.02, 4.60	4.07, 4.65	4.10, 4.68	4.13, 4.71

**Table A2:** Detailed band gap energy ($E_{g}$) of 2 × 2 × 1 wurtzite Zn${}_{1}{}_{-}{}_{x}$Mg${}_{x}$O (WZ-ZMO1) for varied concentration with external pressure from 0 to 10 GPa in 2 GPa increments corresponding to Figure 3b.

x	0 GPa	2 GPa	4 GPa	6 GPa	8 GPa	10 GPa
	PBE, GLLBSC (eV)					
0.125	3.51, 4.11	3.87, 4.46	3.92, 4.51	3.96, 4.56	4.00, 4.60	4.05, 4.64
0.25	2.54, 3.30	4.21, 4.82	4.26, 4.87	3.36, 4.09	3.40, 4.13	3.43, 4.16
0.375	2.69, 3.46	4.52, 5.14	4.57, 5.19	4.61, 5.23	4.66, 5.28	4.69, 5.31
0.5	3.19, 4.00	4.80, 5.44	4.85, 5.49	4.89, 5.53	4.94, 5.57	5.11, 5.73
0.625	4.61, 5.30	5.69, 6.35	5.75, 6.40	5.80, 6.46	5.85, 6.50	5.90, 6.55
0.75	5.32, 6.05	6.16, 6.84	6.21, 6.89	6.27, 6.95	6.33, 7.00	6.38, 7.05
0.875	5.71, 6.50	6.80, 7.04	6.86, 7.59	6.93, 7.64	6.98, 7.70	7.04, 7.75

**Table A3:** Detailed band gap energy ($E_{g}$) of 2 × 2 × 1 wurtzite Zn${}_{1}{}_{-}{}_{x}$Mg${}_{x}$O (WZ-ZMO2) for varied concentration with external pressure from 0 to 10 GPa in 2 GPa increments corresponding to Figure 4b.

x	0 GPa	2 GPa	4 GPa	6 GPa	8 GPa	10 GPa
	PBE, GLLBSC (eV)					
0.125	2.89, 3.74	2.91, 3.77	2.93, 3.79	2.95, 3.81	2.96, 3.82	2.98, 3.84
0.25	3.80, 4.43	3.84, 4.47	3.88, 4.51	3.93, 4.56	3.98, 4.61	2.96, 3.67
0.375	4.35, 4.97	4.39, 5.01	4.44, 5.05	4.48, 5.10	4.53, 5.14	4.57, 5.18
0.5	4.88, 5.52	4.92, 5.55	4.84, 5.48	5.01, 5.64	4.93, 5.57	4.97, 5.61
0.625	5.07, 5.72	5.11, 5.77	5.16, 5.81	5.20, 5.85	5.24, 5.90	5.29, 5.94
0.75	4.12, 5.11	4.16, 5.15	4.18, 5.18	4.21, 5.21	4.24, 5.23	4.27, 5.26
0.875	6.70, 7.44	4.42, 5.47	4.46, 5.51	4.49, 5.53	4.52, 5.57	4.54, 5.59

## Appendix B. Detailed Density of States for x = 0.125, 0.5 and 0.875 with the Increasing External Pressures from 0 to 10 GPa

The complete density of states correspond to the Figure 5 for x = 0.125, 0.5 and 0.875 with the increasing external pressures from 0 to 10 GPa in 2 GPa increments.
